# Compatibility of Nucleobases Containing Pt(II) Complexes with Red Blood Cells for Possible Drug Delivery Applications

**DOI:** 10.3390/molecules28196760

**Published:** 2023-09-22

**Authors:** Federica De Castro, Erika Stefàno, Francesco Paolo Fanizzi, Riccardo Di Corato, Pasant Abdalla, Francesca Luchetti, Maria Gemma Nasoni, Rosaria Rinaldi, Mauro Magnani, Michele Benedetti, Antonella Antonelli

**Affiliations:** 1 Department of Biological and Environmental Sciences and Technologies, University of Salento, Via Monteroni, 73100 Lecce, Italy; federica.decastro@unisalento.it (F.D.C.); erika.stefano@unisalento.it (E.S.); 2 Center for Biomolecular Nanotechnologies, Istituto Italiano di Tecnologia, 73010 Arnesano, Italy; riccardo.dicorato@cnr.it; 3 Institute for Microelectronics and Microsystems (IMM), CNR, Via Monteroni, 73100 Lecce, Italy; ross.rinaldi@unisalento.it; 4 Dipartimento di Scienze Biomolecolari, Università degli Studi di Urbino Carlo Bo, Via Saffi 2, 61029 Urbino, Italy; p.abdalla@campus.uniurb.it (P.A.); francesca.luchetti@uniurb.it (F.L.); maria.nasoni@uniurb.it (M.G.N.); mauro.magnani@uniurb.it (M.M.); 5 Mathematics and Physics “E. De Giorgi” Department, University of Salento, Via Monteroni, 73100 Lecce, Italy

**Keywords:** cisplatin, platinum drugs, coordination compounds, nucleoside analogues, drug delivery systems, red blood cells

## Abstract

The therapeutic advantages of some platinum complexes as major anticancer chemotherapeutic agents and of nucleoside analogue-based compounds as essential antiviral/antitumor drugs are widely recognized. Red blood cells (RBCs) offer a potential new strategy for the targeted release of therapeutic agents due to their biocompatibility, which can protect loaded drugs from inactivation in the blood, thus improving biodistribution. In this study, we evaluated the feasibility of loading model nucleobase-containing Pt(II) complexes into human RBCs that were highly stabilized by four *N*-donors and susceptible to further modification for possible antitumor/antiviral applications. Specifically, platinum-based nucleoside derivatives [Pt^II^(dien)(*N7*-Guo)]^2+^, [Pt^II^(dien)(*N7*-dGuo)]^2+^, and [Pt^II^(dien)(*N7*-dGTP)] (dien = diethylenetriamine; Guo = guanosine; dGuo = 2′-deoxy-guanosine; dGTP = 5′-(2′-deoxy)-guanosine-triphosphate) were investigated. These Pt(II) complexes were demonstrated to be stable species suitable for incorporation into RBCs. This result opens avenues for the possible incorporation of other metalated nucleobases analogues, with potential antitumor and/or antiviral activity, into RBCs.

## 1. Introduction

Research and development on red blood cells (RBCs) as drug delivery systems has increased rapidly in the last twenty years. Due to their long lifespans (approximately 120 days in humans), high surface-to-volume ratios, and non-immunogenicity, RBCs represent a widely used biocompatible method for the delivery of peptides, proteins, small molecules, nucleic acids, antibodies, and a large number of nanomaterials compared to other types of drug delivery systems [1,2,3,4,5,6,7,8,9]. Drugs can be loaded in these cells and subsequently reinfused into the same or matched donors. Nowadays, RBC-based delivery systems have been studied in thousands of infusions in humans, widely demonstrating safety and efficacy [10,11]. Erythrocyte-based therapies have the advantage of keeping the drugs in circulation active, slowly releasing them over several weeks. Moreover, RBCs allow for the targeted release of therapeutic agents in cells or organs, bypassing stability problems in biological fluids, decreasing the amount of drugs, and, consequently, limiting potential side effects [12,13]. As for erythrocytes loaded with antiviral drugs (mainly represented by nucleoside analogues) or antibiotic agents, they can induce in situ inhibition of viruses and microorganisms’ replication [13,14,15,16,17]. Additionally, RBCs are used for diagnostic purposes (e.g., magnetic resonance imaging, etc.) [3,4,5,13,18,19,20,21,22]. Loading of contrast agents in RBCs has been demonstrated to prolong their lifespan in the bloodstream, leading to improvements in biomedical and diagnostic fields [4,23,24,25]. RBC-based delivery systems can also be useful to control certain drug toxicity profiles. As a delivery system, they could control the release of molecules considered to be not very effective or with a certain toxicity profile, improving their pharmacokinetics, bioavailability, and biological activity [13,15]. Due to their interesting properties, RBCs are promising drug carriers, and they have been widely studied for the delivery of peptides, enzymes, and antineoplastic drugs [6,11,12,13,18,19,20]. However, thus far, only a limited number of studies have reported the investigation of RBCs as drug delivery systems, with only a few focused on metal-based drugs [6,26,27,28,29,30,31,32,33,34,35], which include some examples focused on Pt drugs. Despite a huge amount of research on cisplatin-related compounds [36,37,38], the incorporation of platinum drugs in RBCs represents a largely unexplored field. Herein, we report for the first time the possibility to load new model platinated nucleoside analogues in human RBCs.

Metal-based drugs are a class of compounds that have been extensively studied for their wide range of applications, from anticancer [39,40,41] to antiviral applications [42,43] and for several other disorders [38,44]. The development of metallo-drugs for clinical purposes began with the discovery of the antitumor properties of cisplatin, a platinum(II) derivative, and its FDA approval in 1978 [45]. Despite the great success of platinum drugs in the clinic for the treatment of several solid tumors, their therapeutic outcomes are still limited by severe side effects, drug resistance, and poor pharmacokinetic properties, which limit the efficacy of these antitumor agents [37]. Thousands of new platinum drug candidates have been synthesized and studied with the aim of reducing drug resistance and drug-induced side effects, improving oral bioavailability, and overcoming inter-individual variability of responses [42,46,47,48]. Among these, new platinum-based nucleoside analogues derivatives were synthesized [42,43,49,50,51,52]. This new class of compounds has recently been demonstrated to have very interesting properties derived from the combination of those of platinum compounds and nucleoside analogs, with their potential application ranging from antitumor to antiviral use [42]. Anticancer and/or antiviral metalated nucleotide analogues can be considered a potential new generation of metal-based drugs that still need to be studied and further explored.

Recently, we devoted our research to new platinated guanine derivatives in order to gain a deeper understanding of their cellular metabolic mechanisms, and to confirm their potential as antimetabolites [42,52,53,54,55,56,57,58]. The considered compounds demonstrated the ability to be incorporated into newly synthesized DNA (nuclear and mitochondrial) by DNA polymerases, thus mimicking the natural cellular counterparts [54,58]. Furthermore, the considered platinum-based nucleosides are able to cross the cell and mitochondrial membranes by using nucleoside transporters [52,53]. Interestingly, the field of RBCs used as drug delivery systems for antiviral compounds and specifically for nucleobase analogues has been investigated and constitutes a promising research area [16,17,59].

**Scheme 1:**
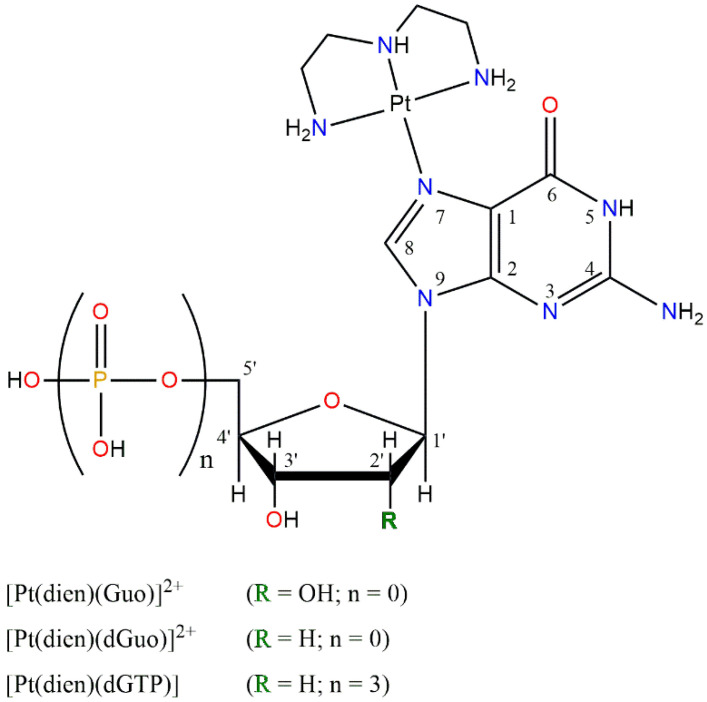
Schematic structure of the square-planar platinum(II) complexes with four *N*-donors considered in this work: [Pt^II^(dien)(*N*7-Guo)]^2+^ (**1**), [Pt^II^(dien)(*N*7-dGuo)]^2+^ (**2**), and [Pt^II^(dien)(*N*7-dGTP)] (**3**) (dien = diethylenetriamine; Guo = guanosine; dGuo = 2′-deoxy-guanosine; dGTP = 5′-(2′-deoxy)-guanosine-triphosphate). Standard atomic numbering is reported, atoms other than C and H are reported with their standard color code. For simplicity, the platinum oxidation state and bonded *N7* have not been indicated in most of the formulae reported in this work for complexes **1**–**3**.

In this context, the present work aims to study whether RBCs can be used as delivery systems for the model platinated nucleoside analogues: [Pt^II^(dien)(*N*7-Guo)]^2+^ (**1**), [Pt^II^(dien)(*N*7-dGuo)]^2+^ (**2**), and [Pt^II^(dien)(*N*7-dGTP)] (**3**) (dien = diethylenetriamine; Guo = guanosine; dGuo = 2′-deoxy-guanosine; and dGTP = 5′-(2′-deoxy)-guanosine-triphosphate). In the following discussion, complexes **1**–**3** (Scheme 1) will be generally referred to, in short, as [Pt(dien)(Guo)]^2+^, [Pt(dien)(dGuo)]^2+^, and [Pt(dien)(dGTP)], respectively. These complexes, despite not being intrinsically active as antitumor or antiviral drugs, are recognized by physiological membrane transporters and DNA polymerases, thus representing suitable models for assessing the stability of RBCs.

Their compatibility with RBC loading may thus provide potential future developments of analogous loadings of similar active platinum complexes based on modified nucleosides. Herein, we report results concerning the biocompatibility tests versus incorporation in human RBCs of platinated nucleoside analogs, with a square-planar coordination geometry and four *N*-donors in the Pt(II) coordination sphere.

## 2. Results and Discussion

### 2.1. Investigation of Biocompatibility and Incorporation of Platinum Complexes in Human Red Blood Cells (RBCs)

**Figure 1:**
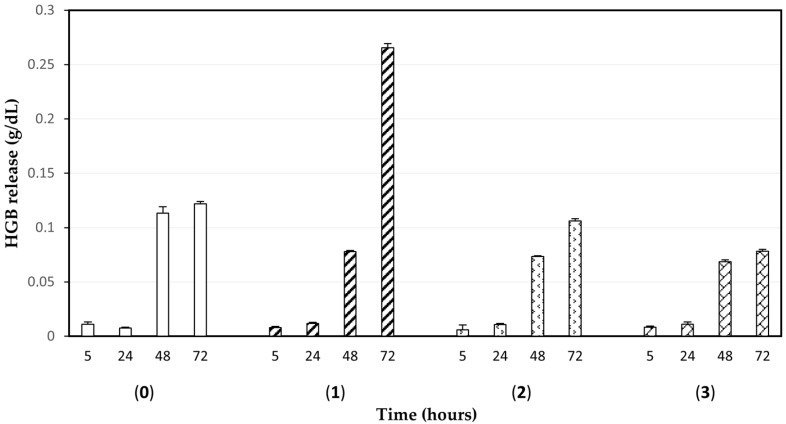
Spectrophotometric evaluation of total hemoglobin (HGB, g/dL) in supernatants derived from erythrocyte suspensions (500 µL) incubated with platinum [Pt^II^(dien)(*N*7-Guo)]^2+^ (**1**), [Pt^II^(dien)(*N*7-dGuo)]^2+^ (**2**), and [Pt^II^(dien)(*N*7-dGTP)] (**3**) complexes at 10 mM concentration and at different time intervals (dien = diethylenetriamine; Guo = guanosine; dGuo = 2′-deoxy-guanosine; dGTP = 5′-(2′-deoxy)-guanosine-triphosphate). (**0**) NT (not treated RBCs).

Before carrying out loading tests into human erythrocytes, we considered it necessary to preliminarily verify the biocompatibility of these platinum compounds with RBCs. Aliquots of each compound [Pt^II^(dien)(*N*7-Guo)]^2+^ (**1**), [Pt^II^(dien)(*N*7-dGuo)]^2+^ (**2**), and [Pt^II^(dien)(*N*7-dGTP)] (**3**) were added to 0.5 mL 44% Ht RBCs at a 10 mM final concentration. The samples were incubated at room temperature and with slight stirring at different times (5, 24, 48, and 72 h). Aliquots of these suspensions were taken to evaluate, through a hemocytometer, some typical biological parameters of human RBCs in comparison to those of the control sample (NT, not treated RBCs). The results reported in Table 1 show that the presence of platinum complexes does not significantly alter the basic values of RBCs. The MCV of RBCs appears to be smaller only after 72 h of incubation, but this occurs for untreated cells as well. To further evaluate the cell integrity after the addition of complexes **1**–**3**, at each time point, supernatants obtained as reported in “Materials and Methods” section were used to evaluate a possible release of hemoglobin compared to the control untreated sample. The data reported in Figure 1 show an increase in the presence of released hemoglobin (HGB) after 48 h that is not significantly different from that observed in the control sample. Although a double amount of hemoglobin appears to be released at 72 h with respect to control (NT) in the case of [Pt(dien)(Guo)]^2+^, in all cases the overall observed HGB released from treated cells (Figure 1) does not significantly affect the HGB content within the cells (Table 1) (HGB released < 2% HGB total RBCs content). Additionally, it is clear that alterations of some hematological parameters may occur in long-time experimental conditions (such as 72 h, Table 1); this appears to be evident mostly for compound **1** when compared to the others.

After carrying out preliminary tests to evaluate the biocompatibility of these specific compounds with human RBCs, we performed the incorporation of the *N7*-platinated guanosine derivatives of the type: [Pt(dien)(Guo)]^2+^ (**1**), [Pt(dien)(dGuo)]^2+^ (**2**), and [Pt(dien)(dGTP)] (**3**) (dien = diethylenetriamine; Guo = guanosine; dGuo = 2′-deoxy-guanosine; dGTP = 5′-(2′-deoxy)-guanosine-triphosphate) using the loading protocol reported in “Materials and Methods” section. The osmolarities of RBCs suspensions were evaluated with a Milliosmometer (OSMOMAT 3000, Gonotec) following the dialysis step and resulting in 110 mOsm for unloaded RBCs; 92 mOsm for (RBCs + **1** sample); 100 mOsm for (RBCs + **2** sample); and 91 mOsm for (RBCs + **3** sample). At the end of the loading procedure, the typical properties of human RBCs were evaluated to verify their cell viability and integrity.

**Figure 2:**
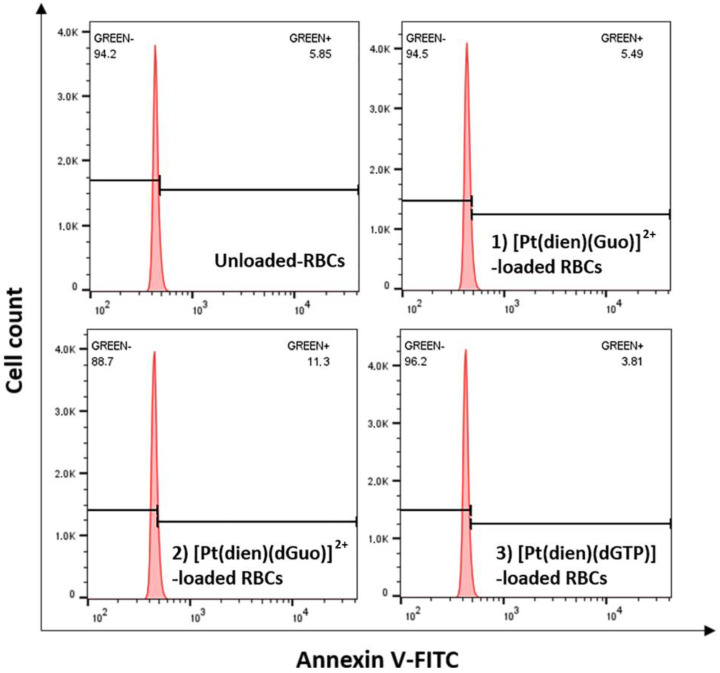
Annexin V binding assay. Representative flow cytometry analysis data from Annexin V-Alexa Fluor 488 staining. The gates in the histogram show the percentage of annexin V negative (green-) and annexin V positive (green+) populations in unloaded RBCs and [Pt^II^(dien)(*N*7-Guo)]^2+^ (**1**), [Pt^II^(dien)(*N*7-dGuo)]^2+^ (**2**), and [Pt^II^(dien)(*N*7-dGTP)] (**3**) complexes-loaded RBCs, five days after preparation (dien = diethylene-triamine; Guo = guanosine; dGuo = 2′-deoxy-guanosine; dGTP = 5′-(2′-deoxy)-guanosine-triphosphate).

Table 2 shows the measurements of the biological characteristics of representative [Pt(dien)(Guo)]^2+^-, [Pt(dien)(dGuo)]^2+^-, and [Pt(dien)(dGTP)]-loaded RBCs samples that do not appear to be altered after the incorporation of all platinum derivatives (**1**, **2**, **3**). In fact, it is evident that the basal values of the control RBCs are not significantly affected after cell incorporation of the platinum compounds. Additionally, the percentage of final cell recoveries is very similar to that obtained for the control sample; only the MCV appears to be slightly lower, as found for the loading of other compounds or molecules [5]. Moreover, in a further investigation of the cell integrity of the Pt complexes-loaded RBCs, possible exposure of phosphatidylserine (PS) on the erythrocyte membrane was evaluated by a specific test. In order to assess the expression of PS on the RBC surface, annexin V binding was measured for samples obtained after the addition of 9 mM platinum complexes in comparison to the control RBCs sample (UL, unloaded), Figure 2. After five days from the preparation of the three platinum compounds-loaded RBCs samples, 200 µL of each sample at 10% of hematocrit was used to analyze Annexin V binding by using the Tali TM image-based cytometer. The percentages of RBCs positively stained with Annexin V were not higher than that found for the control sample (unloaded RBCs, 7%), as shown in Figure 2. These results showed that all Pt complexes-loaded RBC samples present viable cells in similar percentages, which were not significantly different from those of the unloaded RBC sample. This is important, considering that the assay was performed five days after the loaded RBC samples were prepared. In addition, the mean cell diameter of 6 µm was comparable to that of the control cells.

### 2.2. NMR Spectroscopy Determination of Loading Efficiency of Platinated Nucleos(t)ides in RBCs

Nuclear magnetic resonance (NMR) spectroscopy is one of the most widely used analytical techniques for analyzing blood metabolites [60]. ^1^H-NMR spectroscopy is an efficient method for identifying and quantifying complex mixtures in solution [61,62]. This technique has demonstrated great potential as a detection technique, mainly due to its ability to detect multiple (tens to hundreds) metabolites at once without separation [61]. Furthermore, NMR is non-destructive, quantitative, and allows for obtaining the metabolic profiles or “fingerprint” collections of the examined biological samples.

**Figure 3:**
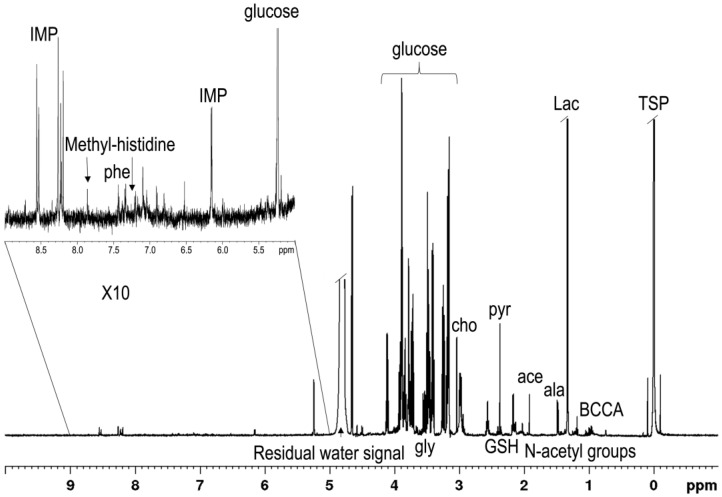
^1^H CPMG NMR spectrum of unloaded red blood cells sample showing the following compounds: BCCA: branched aminoacids; Lac: lactate; ala: alanine; ace: acetate; pyr: pyruvate; GSH: glutathione; cho: choline; gly: glycine; IMP: inositol monophosphate; and phe: phenylalanine.

NMR spectral acquisitions of the supernatants derived from the hemolyzed RBCs loaded samples (with complexes **1**, **2**, and **3**, respectively) were performed to assess the incorporation of the three platinum(II) derivatives in the RBCs. For comparison, the unloaded sample was also considered. Primary resonances in the ^1^H CPMG NMR spectrum of the hemolyzed unloaded RBCs sample were assigned to individual metabolites and are reported in Figure 3.

**Figure 4:**
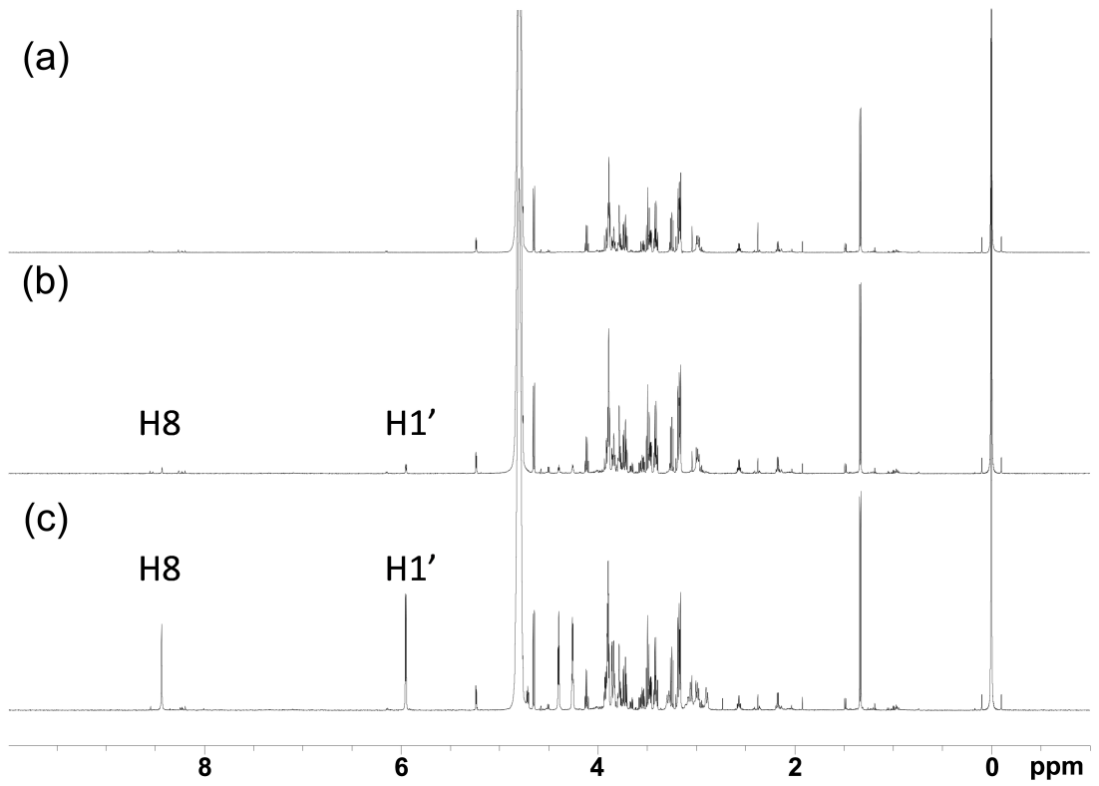
^1^H CPMG NMR spectra of (**a**) unloaded RBCs; (**b**) [Pt^II^(dien)(*N*7-Guo)]^2+^-loaded red blood cells (**1**, dien = diethylenetriamine; Guo = guanosine); and (**c**) addition of complex **1** at a known concentration in the corresponding [Pt(dien)(Guo)]^2+^-loaded RBCs sample.

To reduce protein and water signals, the Carr–Purcell–Meiboom–Gill (CPMG) acquisition sequence with presaturation of the water signal was used. This sequence enabled the clear observation of the signals of the low molecular weight metabolites characterizing the hemolysates and the specific signals of the test complexes. To make the results of each sample comparable, the same acquisition parameters and the same number of scans were used for each sample. In the ^1^H CPMG NMR spectra, the signals of each tested platinum complex were clearly identified (see Figure 4, Figures S1 and S2), demonstrating the successful loading of the complexes in the RBCs. Moreover, the loading procedures did not alter the considered complexes. To determine the concentration of the complexes in the RBCs samples, a quantitative NMR method was used [63,64]. To verify the signals of the compounds and for quantification purposes, complexes **1**, **2** and **3** were added to the tubes at known concentrations (Figure S3) and were also referenced to the signal of the standard TSP. The platinum concentration in the supernatants derived from the hemolyzed RBCs loaded (with complexes **1**, **2** and **3**) and unloaded samples was also analyzed with ICP-AES spectroscopy, providing results similar to those obtained with NMR for the concentrations of complexes **1**, **2**, and **3**.

The concentrations of 1.4_NMR_ (1.6_ICP_), 2.1_NMR_ (1.8_ICP_), and 2.1_NMR_ (2.4_ICP_) mM were determined for the samples 1-3, containing the [Pt(dien)(Guo)]^2+^ (**1**), [Pt(dien)(dGuo)]^2+^ (**2**), and [Pt(dien)(dGTP)] (**3**) complexes, respectively. At the same loading condition, a higher loading efficiency was found for the nucleoside–platinum derivatives [Pt(dien)(dGuo)]^2+^ (**2**), and [Pt(dien)(dGTP)] (**3**) than for the nucleotide–platinum derivative [Pt(dien)(Guo)]^2+^ (**1**).

### 2.3. Cell Integrity Check of Loaded RBCs and Drug Biocompatibility Remarks

**Figure 5:**
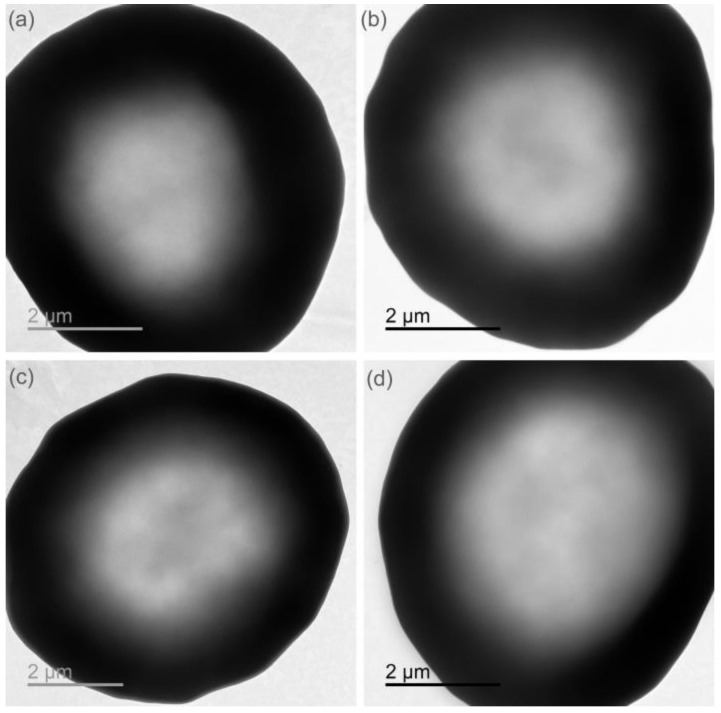
Transmission electron microscopy (TEM) analysis of (**a**) unloaded, (**b**) [Pt^II^(dien)(*N*7-Guo)]^2+^-loaded, (**c**) [Pt^II^(dien)(*N*7-dGuo)]^2+^-loaded, and (**d**) [Pt^II^(dien)(*N*7-dGTP)]-loaded red blood cells. Additional TEM characterization of the loaded RBCs is reported in Figure S4 (single RBCs) and Figure S5 (multiple RBCs).

Through whole-cell TEM analyses, the cell integrity of loaded RBCs was investigated (Figure 5). Results obtained after the loading of RBCs with [Pt(dien)(Guo)]^2+^, [Pt(dien)(dGuo)]^2+^, and [Pt(dien)(dGTP)] (Figure 5b–d, respectively), compared to the control sample (Figure 5a, unloaded RBCs), indicated that the platinum complexes do not appear to affect the RBCs integrity, as no evident alterations in erythrocyte membrane structure were observed.

The goal of this preliminary research was to determine the potential use of these new platinated nucles(t)ides through a biomimetic drug delivery, such as that offered by the most common natural cells, the RBCs. Indeed, erythrocytes are biocompatible, have a large capacity, and can accommodate traditional chemical entities, biologics (proteins), and/or contrasting agents (dyes and nanoparticles). The tested new Pt(II) derivatives were found to be biocompatible and encapsulable in human RBCs, thus supporting the primary aim of the present study. These data could be important in the perspective of using autologous RBCs–platinum compound preparations as therapeutic agents. We have previously reported that drug-carrier RBCs obtained with our procedure can be perfused in the blood system without affecting the other cell components, as their behavior is similar to that of endogenous circulating erythrocytes. After an average survival of about 120 days, RBCs are degraded by the cells of the reticuloendothelial system (i.e., monocytes and macrophages of the spleen and liver).

The data reported in this work showed that the hematological values of human [Pt(dien)(Guo)]^2+^ (**1**), [Pt(dien)(dGuo)]^2+^ (**2**), and [Pt(dien)(dGTP)] (**3**) complexes-loaded RBCs are similar to each other and to the control sample. Thus, the incorporation of these Pt(II) complexes with four *N*-donors, including a nucleos(t)ide, into human RBCs is possible, with a final cell recovery very similar to that of unloaded control cells. These data must be considered as a demonstration of effective biocompatibility. All three compounds have demonstrated that they do not affect the biological properties of RBCs even after the loading procedure. Cell integrity and morphology were also maintained, as shown by TEM analyses.

Red blood cells, if considered as drug carriers, have the potential to protect the body from drug toxicity, greatly reducing side effects and thus increasing the tolerability of pharmacological treatments. This could be an important focus to pursue for the platinum-based antitumor drugs, which generally produce a wide spectrum of side effects mediated by undesired interactions with serum and other proteins. For this reason, in this work, we tested Pt(II)-based complexes, which, as recently experimentally shown [52,53,54,55,58], are able to interfere with nucleoside metabolism. This action mechanism generally occurs for a variety of well-known antitumor and antiviral drugs [42]. We chose complexes **1**–**3** for our studies on biocompatibility with erythrocytes, also due to their low reactivity, being coordinatively stable, due to the presence of four *N*-donors, including a guanine nucleoside derivative, *N*-bonded to platinum. Biocompatibility with RBCs should be enhanced in these complexes due to the absence of a nucleus or other DNA-containing organelles and even for the well-known reduced guanosine metabolism occurring in RBCs [65].

## 3. Materials and Methods

### 3.1. Reagents and Methods

All solvents and reagents, unless otherwise stated, were purchased from Aldrich Chemical Company and used as received. NMR spectra were recorded on a Bruker AVANCE III 400 NMR spectrometer operating at 400.13 Hz, equipped with a probe for inverse detection and a z-gradient for gradient-accelerated NMR spectroscopy. The NMR spectra were recorded at a temperature of 300 K, using D_2_O as the solvent. ^1^H and ^31^P NMR spectra were referenced to the residual HOD signal (δ(^1^H) = 4.7 ppm) and 85% H_3_PO_4_ (δ(^31^P) = 0.0 ppm), respectively, as the internal and external standards.

### 3.2. Synthesis of [Pt(dien)(N7-G)] Complexes

[PtCl(dien)]Cl was prepared following the synthesis reported by Annibale and co-workers [66]. [Pt^II^(dien)(*N*7-Guo)]^2+^ (**1**), [Pt^II^(dien)(*N*7-dGuo)]^2+^ (**2**), and [Pt^II^(dien)(*N*7-dGTP)] (**3**) complexes (dien = diethylenetriamine; Guo = guanosine; dGuo = 2′-deoxy-guanosine; dGTP = 5′-(2′-deoxy)-guanosine-triphosphate) were synthesized by a previously reported method [54,55]. In a typical reaction, the stoichiometric amount of D_2_O solutions of Guo, dGuo, or dGTP at pH ≈ 3, 3, and 5, respectively (adjusted by addition of DCl), was gradually added (1:1 molar ratio) to an approximately 100 mM mother D_2_O solution of the [PtCl(dien)]Cl complex. The reaction was then monitored using ^1^H NMR spectroscopy. The D_2_O solutions containing the final **1**–**3** species were then lyophilized, to eliminate the deuterated solvent and replace it with H_2_O. NMR (400 MHz, D_2_O) (**1**) δ(^1^H, pH ≈ 3) 8.44 (s, 1H, CH), 5.98 (d, ^3^*J*_H-H_ = 5.3 Hz, 1H, CH), 4.73 (t, ^3^*J*_H-H_ = 5.3 Hz, 1H, CH), 4.42 (t, ^3^*J*_H-H_ = 4.6 Hz, 1H, CH), 4.28 (m, 1H, CH), 3.90 (m, 2H, CH), 3.30 (m, 2H, CH), 3.08 (m, 4H, CH), 2.91 ppm (m, 2H, CH); (**2**) δ(^1^H, pH ≈ 3) 8.43 (s, 1H, CH), 6.36 (t, ^3^*J*_H-H_ = 6.6 Hz, 1H, CH), 4.64 (m, 1H, CH), 4.18 (m, 1H, CH), 3.83 (m, 2H, CH), 3.30 (pd, 2H, CH), 3.08 (m, 4H, CH), 2.91 (pd, 2H, CH), 2.78 (m, 1H, CH), 2.58 ppm (m, 1H, CH); (**3**) δ(^1^H, pH ≈ 5) (s, 1H, CH), 6.39 (t, ^3^*J*_H-H_ = 6 Hz, 1H, CH), 4.92 (m, 1H, CH), 4.78 (m, 2H, CH), 4.26 (m, 3H, CH), 3.32 (m, 1H, CH), 3.12 (m, 4H, CH), 2.89 (m, 1H, CH), 2.74 (m, 1H, CH), 2.62 ppm (m, 1H, CH); δ(^31^P, pH = 5) −11.99 (d, ^2^*J*_P-P_ = 52 Hz, 1P; γ-PO_4_), −12.18 (d, ^2^*J*_P-P_ = 48 Hz, 1P; α-PO_4_), −24.15 ppm (t, ^2^*J*_P-P_ = 48 Hz, 1P; β-PO_4_).

### 3.3. Evaluation of the Biocompatibility of [Pt(dien)(Guo)]^2+^ (**1**), [Pt(dien)(dGuo)]^2+^ (**2**), and [Pt(dien)(dGTP)] (**3**) Complexes with Red Blood Cells (RBCs)

For the three platinum complexes used for reported biocompatibility tests with human RBCs, we used starting mother solutions with the following concentrations: (**1**) = 89.1, (**2**) = 78.7, and (**3**) = 61.8 mM. We obtained heparinized blood from the Transfusion Centre of the “*S. Maria della Misericordia*” Hospital in Urbino (PU), Italy, and removed the plasma and buffy coat by washing the sample in Hepes buffer (10 mM Hepes, 140 mM NaCl, 5 mM glucose, pH 7.4) and centrifuging at 1400× *g* at 4 °C. We added the platinum complexes to the RBCs at 44% hematocrit (Ht) to obtain a 10 mM final concentration. We incubated the cell suspensions at room temperature for different times (5, 24, 48, and 72 h) and evaluated the typical biological parameters of RBCs using a hemocytometer (ABX, HORIBA, Montpellier, France) in comparison to a control RBC sample. The Pt-RBCs incubation was performed at room temperature (~20 °C) to prevent oxidation of the RBCs, involving the heme group, taking also into account the usage temperature limits recommended for blood derivatives [67]. To assess cell integrity, hemoglobin (HGB) released in the supernatants obtained after centrifuging the RBC suspensions (containing platinum complexes) at 1400× *g* and 4 °C was spectrophotometrically measured at 540 nm using the Drabkin’s Reagent (Sigma-Aldrich Italia S.R.L., Milano, Italy). At the end of each time point, each RBC suspension (500 µL) was diluted (1:21) with Hepes buffer and immediately centrifuged at 1400× *g* (for 10 min, at 4 °C); 400 µL of the supernatant were used to obtain the HGB measurement data, which were reported in g/dL compared to the results from untreated RBCs (NT).

### 3.4. Incorporation of Platinum-Nucleos(t)ide Complexes in Human RBCs

Human blood was collected from healthy donors using heparinized tubes. Erythrocytes were isolated by centrifugation at 1400× *g* at 4 °C for 10 min from freshly drawn blood. The serum and buffy coat were removed, and the RBC pellet was washed three times with Hepes buffer and then resuspended in the same buffer at 70% of hematocrit. The cells were dialyzed for 1 h in the presence of [Pt(dien)(Guo)]^2+^, [Pt(dien)(dGuo)]^2+^, or [Pt(dien)(dGTP)] at a final concentration of 9 mM, using a tube with a 12-14 kDa cut-off as reported in ref. [3]. The osmolarity of RBCs (300 mOsm in physiological condition) was evaluated after the dialysis step performed in the presence of the three Pt complexes with a Milliosmometer (OSMOMAT 3000, Gonotec, Berlin, Germany). The resealed cells were recovered by centrifugation at 400× *g* and washed four times with Hepes buffer to remove all the untrapped platinum complexes. Following the same procedure, unloaded erythrocytes (unloaded RBCs) were prepared, without the addition of the complexes. Some biological parameters such as the mean cell volume (MCV), mean hemoglobin concentration (MCH), and mean corpuscular hemoglobin concentration (MCHC) were examined to evaluate cell integrity as previously reported [2,3,4,5].

### 3.5. Annexin V Binding Assay

Annexin V was used as a probe to detect cells that expressed phosphatidylserine (PS) on the cell surface, an event consequent to membrane components in/out translocation. Briefly, the cell pellet was resuspended with 100 μL of Annexin V-Binding Buffer (ABB) and incubated with 5 μL of Annexin V–Alexa Fluor™ 488 conjugate for 20 min. Annexin V-positive RBCs were detected using the Tali™ image-based cytometer (ThermoFisher, Scientific, Waltham, MA USA), and the data were analyzed with FlowJo™ v10 Software.

### 3.6. ICP-AES Measurements

Concentrations of Pt were measured using the Inductively Coupled Plasma Atomic Emission Spectroscopy (ICP-AES). Blood samples were mixed with 200 µL of H_2_O_2_ and 500 µL of 69% superpure HNO_3_, then heated at 100 °C for 10 min in a water bath. The samples were then cooled, diluted with superpure water to a final volume of 5 mL, filtered through syringe filters (pore size 0.22 µm), and measured for Pt content using a Thermo Scientific iCap 7000 Series ICP-AES spectrometer. The spectrometer was previously calibrated for quantitative analysis with four standard solutions containing known concentrations of Pt: 0.001, 0.01, 0.1, and 1.0 mg/L. The calibration line showed a correlation coefficient (r) greater than 0.99 for the measured element. The results were the average of three different measurements, and the Pt final concentrations were expressed as mg/L.

### 3.7. NMR Measurements for Determining the Content of Platinum Complexes **1**–**3**

All measurements were performed on a Bruker AVANCE III 600 Ascend NMR spectrometer (Bruker, Ettlingen, Germany), operating at 600.13 MHz for ^1^H observation, equipped with a TCI cryoprobe incorporating a *z*-axis gradient coil and automatic tuning/matching. All NMR spectra were acquired at 300 K and referenced to the trimethylsilyl propionate (TSP) signal (*δ* = 0.00 ppm). For each sample, a standard 1D ^1^H Carr–Purcell–Meiboom–Gill (CMPG) spin-echo sequence was acquired with 64 transients, 16 dummy scans, a 5 s relaxation delay, a size of FID (free induction decay) of 32K data points, and zero-filling by a factor of 2 to give 64K frequency domain data points, P1 11.38 μs, a spectral width of 12,019.230 Hz (20.0276 ppm), an acquisition time of 1.36 s, a total spin–spin relaxation delay of 1.2 ms, and solvent signal saturation during the relaxation delay. To prepare the NMR tubes, 250 μL of unloaded (sample 0) and loaded (samples 1, 2, and 3 loaded with compounds **1**, **2**, and **3**, respectively) hemolysates dissolved in D_2_O were used. Specific standard addition methods suitable for CPMG spectra [63,64] were used for the evaluation of complexes **1**–**3** in the RBCs lysates. The NMR spectra were processed using Topspin 3.6.1 (Bruker, BioSpin, Milano, Italy) for visual inspection.

### 3.8. TEM Analysis

The integrity of unloaded and loaded RBCs was studied using a transmission electron microscope (TEM) JEOL JEM-1011 operating at an accelerating voltage of 100 kV. TEM analysis on RBC samples was performed after their fixation with 2.5% glutaraldehyde, as previously reported [3]. For the TEM analysis of whole RBCs, the cells were cast onto a polymer-coated grid and embedded in an ultrathin polysaccharide film. In detail, 6–7 µL of glutaraldehyde-fixed RBCs (1–4% Ht) were dropped onto a formvar-coated copper grid and left to react for at least 5 min; after that, the grid was washed twice upside down on a 50 μL drop of water, for 1 min; then, the grid was positioned again upside down on a 50 μL drop of cold carboxymethyl-dextran (10 mg/mL, in water) for 5–10 min; finally, the excess liquid was removed by capillarity with a Whatman filter paper (type 1). The grid was left to dry for 5 min at room temperature and stored for the subsequent analysis.

## 4. Conclusions

This work represents the first study testing the biocompatibility of Pt(II) complexes, with four coordinated *N*-donors, including a single *N7*-bonded purine nucleos(t)ide ligand for incorporation into RBCs. The model system considered here consists of [Pt^II^(dien)(*N*7-Guo)]^2+^ (**1**), [Pt^II^(dien)(*N*7-dGuo)]^2+^ (**2**), and [Pt^II^(dien)(*N*7-dGTP)] (**3**) complexes incorporated into erythrocytes, to be considered for further future delivery studies. The choice of RBCs as a drug delivery system was considered due to their natural role as carriers and components of the blood circulatory system. RBC carrier infusions in humans have been previously reported, proving their safety and efficacy [68]. These biocompatible carriers are thus gaining interest in new diagnostic approaches and in the management of complex pathologies, particularly for drugs that have severe cytotoxicity and/or induce drug resistance [3,4,13,21,24].

The obtained results revealed that Pt(II)-nucleoside analogues adducts can be fully compatible with red blood cells. Preliminary tests of adding compounds **1**–**3** to RBCs allowed us to evaluate the maintenance of normal cell biological features, without a significant effect on the typical hemocytometric parameters. Moreover, the loading of platinum complexes in human RBCs was successfully achieved by using a method of hypotonic dialysis previously reported by Antonelli et al. [4]. The data showed an encapsulation of platinum complexes in human RBCs ranging from 0.2 to 0.4 mM when 9 mM concentration of platinum complexes (**1**–**3**) was used during the loading procedure. It was also found that processed erythrocytes substantially maintained their fundamental properties and cell integrity. Moreover, the Annexin V binding assay showed that no significant phosphatidylserine RBCs surface exposure occurred after encapsulation of platinum complexes into cells with our loading method. Good cell integrity was also supported by transmission electron microscopy (TEM), which showed that the use of this type of platinum compounds does not affect the typical blood cells morphology.

Due to the great interest in the study of platinum-based drugs and the frequent side effects occurring after treatments, our results could open new perspectives for the study of RBCs as possible new delivery systems for platinum-based drugs. This cell type has previously been evaluated for its suitability as a platinum drug carrier, but it showed decreased compatibility with the utilized platinum drugs [6,30,31]. For this reason, in this work, we have considered for the first time the possibility of carrying some platinum complexes with reduced reactivity for the presence of four platinum-bonded *N*-donors, including an *N7*-bonded guanosine derivative. The biological interactions of these molecules were experimentally evaluated by us in the past years toward DNA and RNA polymerases and cell and mitochondrial membrane transporters [42,52,53,54,55,56,57,58]. Complexes **1**–**3**, though not active as antitumor/antiviral drugs, have been used as specific models for their assessed recognition by transporters and potential incorporation into DNA. We previously reported the potential of this class of molecules in developing new antitumor and antiviral drugs [69]. The platinum compounds presented in this work, which are not intrinsically active as antitumor or antiviral drugs, represent suitable models to evaluate prior to searching for new active species specifically tailored for RBC delivery.

In conclusion, we have demonstrated the possibility of loading platinated nucleoside analogues with a square-planar coordination geometry and four *N*-donors in the Pt(II) coordination sphere within human RBCs. Overall, we can conclude that these new nucleobase mimetic molecular species are compatible with red blood cells when used as drug delivery carriers.

## Figures and Tables

**Table 1:** Measurements of hematological values of RBCs in the presence of [Pt^II^(dien)(*N*7-Guo)]^2+^ (**1**), [Pt^II^(dien)(*N*7-dGuo)]^2+^ (**2**), and [Pt^II^(dien)(*N*7-dGTP)] (**3**) complexes at 10 mM concentration and at 5, 24, 48, and 72 h after treatment (dien = diethylenetriamine; Guo = guanosine; dGuo = 2′-deoxy-guanosine; dGTP = 5′-(2′-deoxy)-guanosine-triphosphate).

**5 h**	RBCs 10^6^/μl	HGB g/dL	HCT %	MCV fl	MCH pg	MCHC g/dL
(0) 500 µL RBCs (NT)	5.30 ± 0.1	15.2 ± 0.55	44.6 ± 045	87 ± 1.0	31.6 ± 0.47	33.9 ± 1.4
(1) 500 µL RBCs + **1**	4.87 ± 0.25	15.8 ± 0.30	44.3 ± 0.47	87 ± 1.0	30.9 ± 0.4	35.3 ± 0.32
(2) 500 µL RBCs + **2**	4.67 ± 0.26	13.5 ± 0.4	39.7 ± 0.3	85 ± 0.58	30.7 ± 0.9	35.2 ± 0.55
(3) 500 µL RBCs + **3**	4.85 ± 0.12	14.4 ± 0.4	40.7 ± 0.83	87 ± 0.5	29.9 ± 0.15	34.7 ± 0.46
**24 h**						
(0) 500 µL RBCs (NT)	5.11 ± 0.08	15.4 ± 0.1	44.3 ± 0.47	87 ± 1.0	30.7 ± 0.5	35.1 ± 0.1
(1) 500 µL RBCs + **1**	4.88 ± 0.07	14.2 ± 0.25	44.5 ± 0.4	85 ± 0.57	30.3 ± 0.61	35.3 ± 0.3
(2) 500 µL RBCs + **2**	4.64 ± 0.07	13.9 ± 0.4	39.7 ± 0.37	85 ± 1.0	29.8 ± 0.1	34.7 ± 0.55
(3) 500 µL RBCs + **3**	4.41 ± 0.06	13.3 ± 0.3	37.7 ± 0.32	86 ± 0.57	29.5 ± 0.15	36.8 ± 0.36
**48 h**						
(0) 500 µL RBCs (NT)	5.58 ± 0.09	16.9 ± 0.2	46.6 ± 1.25	85 ± 0.57	30.5 ± 0.45	35.1 ± 0.8
(1) 500 µL RBCs + **1**	4.72 ± 0.16	14.6 ± 0.38	38.9 ± 0.15	83 ± 0.57	30.5 ± 0.58	36.4 ± 0.25
(2) 500 µL RBCs + **2**	4.69 ± 0.1	13.5 ± 0.3	39.5 ± 0.23	83 ± 0.57	29.4 ± 0.15	35.1 ± 0.3
(3) 500 µL RBCs + **3**	3.87 ± 0.13	11.5 ± 0.34	31.7 ± 0.45	83 ± 0.58	30.7 ± 0.35	36.8 ± 0.36
**72 h**						
(0) 500 µL RBCs (NT)	4.35 ± 0.61	12.7 ± 0.45	32.6 ± 0.77	79 ± 1.00	30.7 ± 0.25	37.4 ± 0.81
(1) 500 µL RBCs + **1**	3.85 ± 0.10	13.4 ± 0.21	26.9 ± 0.83	70 ± 0.58	34.7 ± 0.5	49.7 ± 0.8
(2) 500 µL RBCs + **2**	3.64 ± 0.12	10.6 ± 0.2	32.1 ± 0.1	66 ± 1.00	32.3 ± 0.3	47.7 ± 1.6
(3) 500 µL RBCs + **3**	1.38 ± 0.06	3.93 ± 0.25	9.7 ± 0.4	71 ± 1.00	29.8 ± 0.2	41.9 ± 0.25

RBCs, red blood cells; HGB, hemoglobin; HCT, hematocrit; MCV, mean corpuscular volume; MCH, mean corpuscular hemoglobin; MCHC, mean corpuscular hemoglobin concentration.

**Table 2:** Evaluation of hematological values of human [Pt^II^(dien)(*N*7-Guo)]^2+^ (**1**), [Pt^II^(dien)(*N*7-dGuo)]^2+^ (**2**), and [Pt^II^(dien)(*N*7-dGTP)] (**3**) complexes-loaded RBCs (dien = diethylenetriamine; Guo = guanosine; dGuo = 2′-deoxy-guanosine; dGTP = 5′-(2′-deoxy)-guanosine-triphosphate).

Samples	RBCs 10^6^/μL	HGB g/dL	HCT %	MCV fl	MCH pg	MCHC g/dL	Cell Recovery %
ND (Not dialyzed RBCs)	5.84 ± 0.1	15.8 ± 0.3	41.1 ± 1.2	82 ± 0.5	27.0 ± 0.1	38.3 ± 0.21	/
(0) Unloaded RBCs	6.46 ± 0.22	14.3 ± 0.5	41.6 ± 1.3	72 ± 0.6	22.2 ± 0.3	33.9 ± 0.80	68 ± 1.5
(1) RBCs loaded by **1**	6.69 ± 0.10	14.7 ± 0.4	44.6 ± 0.15	69 ± 1.0	22.1 ± 0.2	32.1 ± 0.51	64 ± 1.3
(2) RBCs loaded by **2**	6.87 ± 0.04	14.6 ± 0.1	44.2 ± 0.31	64 ± 0.6	21.5 ± 0.4	33.3 ± 0.57	64 ± 0.58
(3) RBCs loaded by **3**	6.45 ± 0.02	13.4 ± 0.2	42.9 ± 0.85	66 ± 1.7	20.8 ± 0.3	31.4 ± 1.0	65 ± 1.0

RBCs, red blood cells; HGB, hemoglobin; HCT, hematocrit; MCV, mean corpuscular volume; MCH, mean corpuscular hemoglobin; MCHC, mean corpuscular hemoglobin concentration.

## Data Availability

All data are contained within the article and the Supporting Information.

## Supplementary Materials

The following supporting information can be downloaded at: https://www.mdpi.com/article/10.3390/molecules28196760/s1. Figure S1: ^1^H CPMG NMR spectra of (a) unloaded red blood cells (RBCs); (b) [Pt^II^(dien)(*N7*-dGuo)]^2+^-loaded RBCs (**2**, dien = diethylenetriamine; dGuo = 2′-deoxy-guanosine); and (c) addition of complex **2** at a known concentration in the corresponding [Pt(dien)(dGuo)]^2+^-loaded RBCs sample. Figure S2: ^1^H CPMG NMR spectra of (a) unloaded red blood cells; (b) [Pt^II^(dien)(*N7*-dGTP)]-loaded RBCs (**3**, dien = diethylenetriamine; dGTP = 5′-(2′-deoxy)-guanosine-triphosphate); and (c) addition of complex **3** at a known concentration in the corresponding [Pt(dien)(dGTP)]^2+^-loaded RBCs sample. Figure S3: ^1^H CPMG NMR spectra of unloaded and loaded red blood cells samples after the addition of known concentrations of [Pt^II^(dien)(*N7*-Guo)]^2+^ (**1**), [Pt^II^(dien)(*N7*-dGuo)]^2+^ (**2**), and [Pt^II^(dien)(*N7*-dGTP)] (**3**) (dien = diethylenetriamine; Guo = guanosine; dGuo = 2′-deoxy-guanosine; dGTP = 5′-(2′-deoxy)-guanosine-triphosphate; in this figure, for formulae of complexes **1**–**3**, the indication of platinum oxidation state and bonded *N7* have been omitted for simplicity). Figure S4: Transmission electron microscopy (TEM) analysis of (a) unloaded, (b) [Pt^II^(dien)(*N7*-Guo)]^2+^- (c) [Pt^II^(dien)(*N7*-dGuo)]^2+^-, and (d) [Pt^II^(dien)(*N7*-dGTP)]-loaded red blood cells (dien = diethylenetriamine; Guo = guanosine; dGuo = 2′-deoxy-guanosine; dGTP = 5′-(2′-deoxy)-guanosine-triphosphate; in this figure, for formulae of complexes **1**–**3**, the indication of platinum oxidation state and bonded *N7* have been omitted for simplicity). Figure S5: Transmission electron microscopy (TEM) analysis of (a) unloaded, (b) [Pt^II^(dien)(*N7*-Guo)]^2+^- (c) [Pt^II^(dien)(*N7*-dGuo)]^2+^-, and (d) [Pt^II^(dien)(*N7*-dGTP)]-loaded red blood cells (dien = diethylenetriamine; Guo = guanosine; dGuo = 2′-deoxy-guanosine; dGTP = 5′-(2′-deoxy)-guanosine-triphosphate; in this figure, for the formulae of complexes **1**–**3**, the indication of platinum oxidation state and bonded *N7* have been omitted for simplicity).

## Sample Availability

Samples of complexes **1**–**3** are available from authors.
