# Supplementary material for: Compatibility of Nucleobases Containing Pt(II) Complexes with Red Blood Cells for Possible Drug Delivery Applications
Source: Molecules. 2023 Sep 22;28(19):6760. doi: 10.3390/molecules28196760 (PMC10574024; doi:10.3390/molecules28196760)
Supplement: Supplementary file 1 [file molecules-28-06760-s001.zip › molecules-2622989-supplementary.pdf]

Supporting Information

Compatibility of Nucleobases Containing Pt(II) Complexes with Red Blood Cells for Possible Drug Delivery Applications

Federica De Castro [1], Erika Stefàno [1], Francesco Paolo Fanizzi [1,*], Riccardo Di Corato [2,3], Pasant Abdalla [4], Francesca Luchetti [4], Maria Gemma Nasoni [4], Rosaria Rinaldi [3,5], Mauro Magnani [4], Michele Benedetti [1,*] and Antonella Antonelli [4,*]

[1] Department of Biological and Environmental Sciences and Technologies, University of Salento, Via Monteroni, 73100 Lecce, Italy; federica.decastro@unisalento.it (F.D.C.); erika.stefano@unisalento.it (E.S.)
[2] Center for Biomolecular Nanotechnologies, Istituto Italiano di Tecnologia, 73010 Arnesano, Italy; riccardo.dicorato@cnr.it
[3] Institute for Microelectronics and Microsystems (IMM), CNR, Via Monteroni, 73100 Lecce, Italy; ross.rinaldi@unisalento.it
[4] Dipartimento di Scienze Biomolecolari, Università degli Studi di Urbino Carlo Bo, Via Saffi 2, 61029 Urbino, Italy; p.abdalla@campus.uniurb.it (P.A.); francesca.luchetti@uniurb.it (F.L.); maria.nasoni@uniurb.it (M.G.N.); mauro.magnani@uniurb.it (M.M.)
[5] Mathematics and Physics "E. De Giorgi" Department, University of Salento, Via Monteroni, 73100 Lecce, Italy
* Correspondence: fp.fanizzi@unisalento.it (F.P.F.); michele.benedetti@unisalento.it (M.B.); antonella.antonelli@uniurb.it (A.A.)

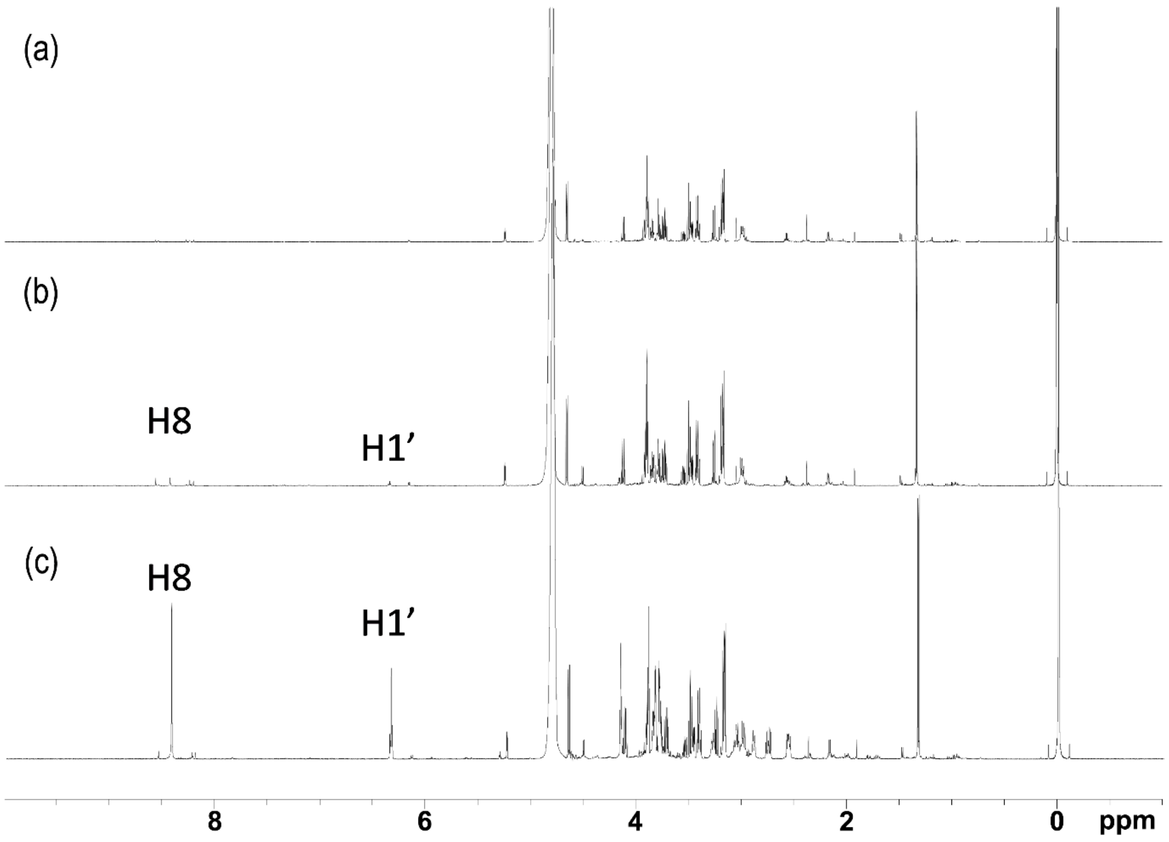

Figure S1. ^{1}H CPMG NMR spectra of: (a) unloaded Red Blood Cells (RBCs); (b) [Pt(dien)(dGuo)]$^{2+}$-loaded RBCs (**2**, dien = diethylenetriamine; dGuo = 2'-deoxy-guanosine); (c) Addition of complex **2** at known concentration in the corresponding [Pt(dien)(dGuo)]$^{2+}$-loaded RBCs sample.

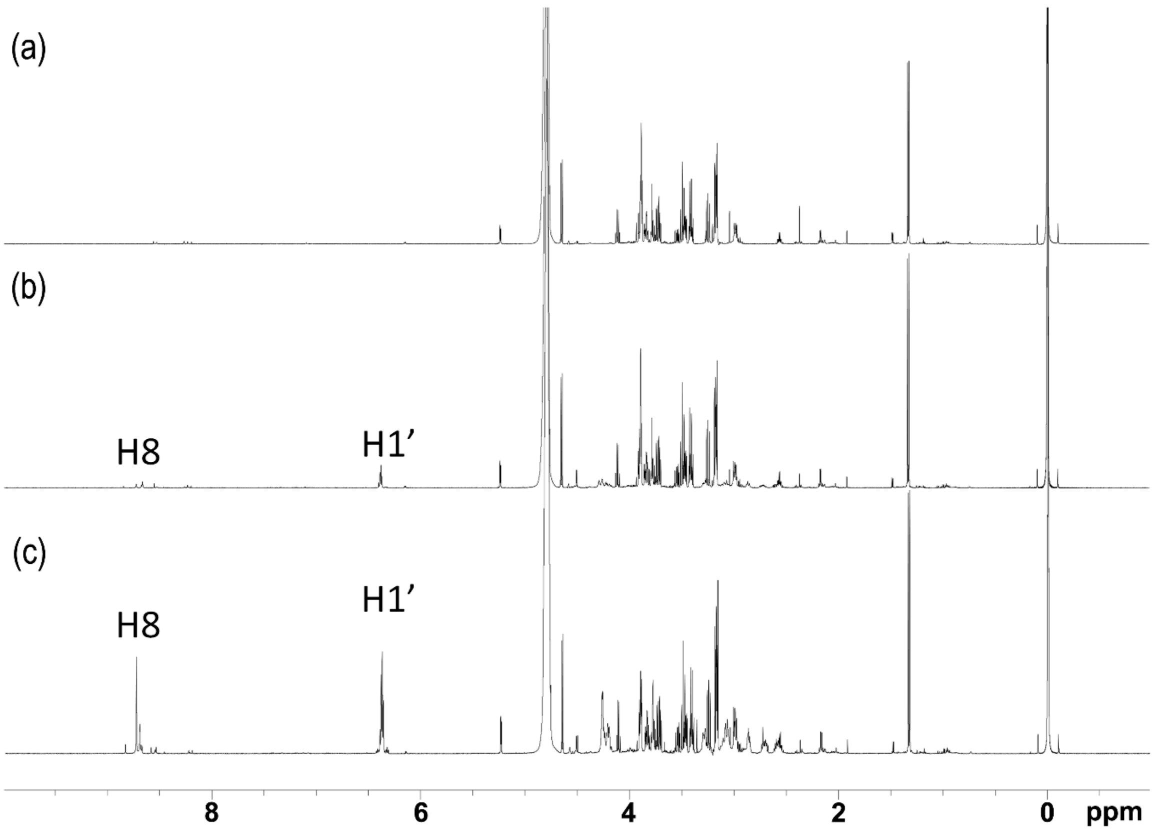

Figure S2. ^{1}H CPMG NMR spectra of: (a) unloaded Red Blood Cells (RBCs); (b) $[Pt(dien)(dGTP)]^{2+}$-loaded RBCs (**3**, dien = diethylenetriamine; dGTP = 5′-(2′-deoxy)-guanosine-triphosphate); (c) Addition of complex **3** at known concentration in the corresponding $[Pt(dien)(dGTP)]^{2+}$-loaded RBCs sample.

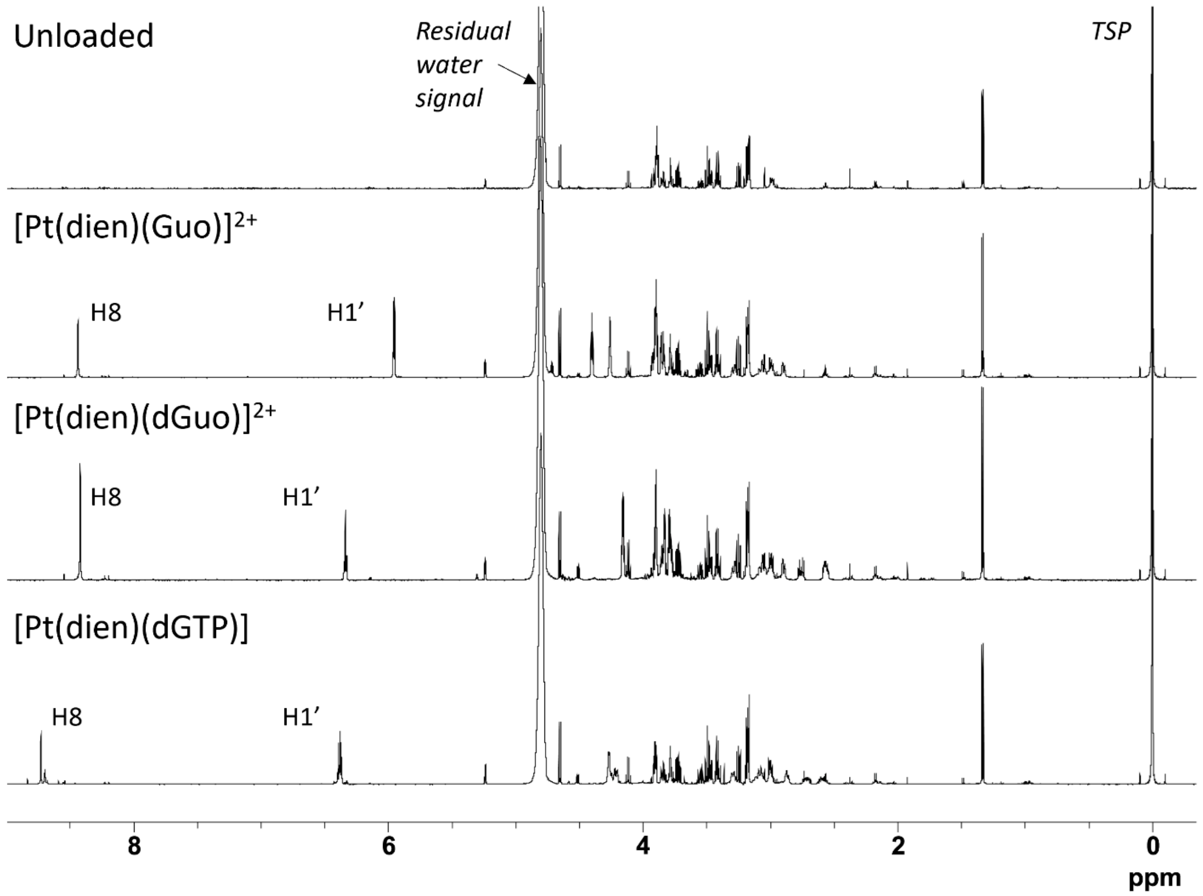

Figure S3. ^1H CPMG NMR spectra of unloaded and loaded Red Blood Cells samples after the addition of known concentrations of $[Pt(dien)(Guo)]^{2+}$ (**1**), $[Pt(dien)(dGuo)]^{2+}$ (**2**), and [Pt(dien)(dGTP)] (**3**) (dien = diethylenetriamine; Guo = guanosine; dGuo = 2’-deoxy-guanosine; dGTP = 5′-(2′-deoxy)-guanosine-triphosphate).

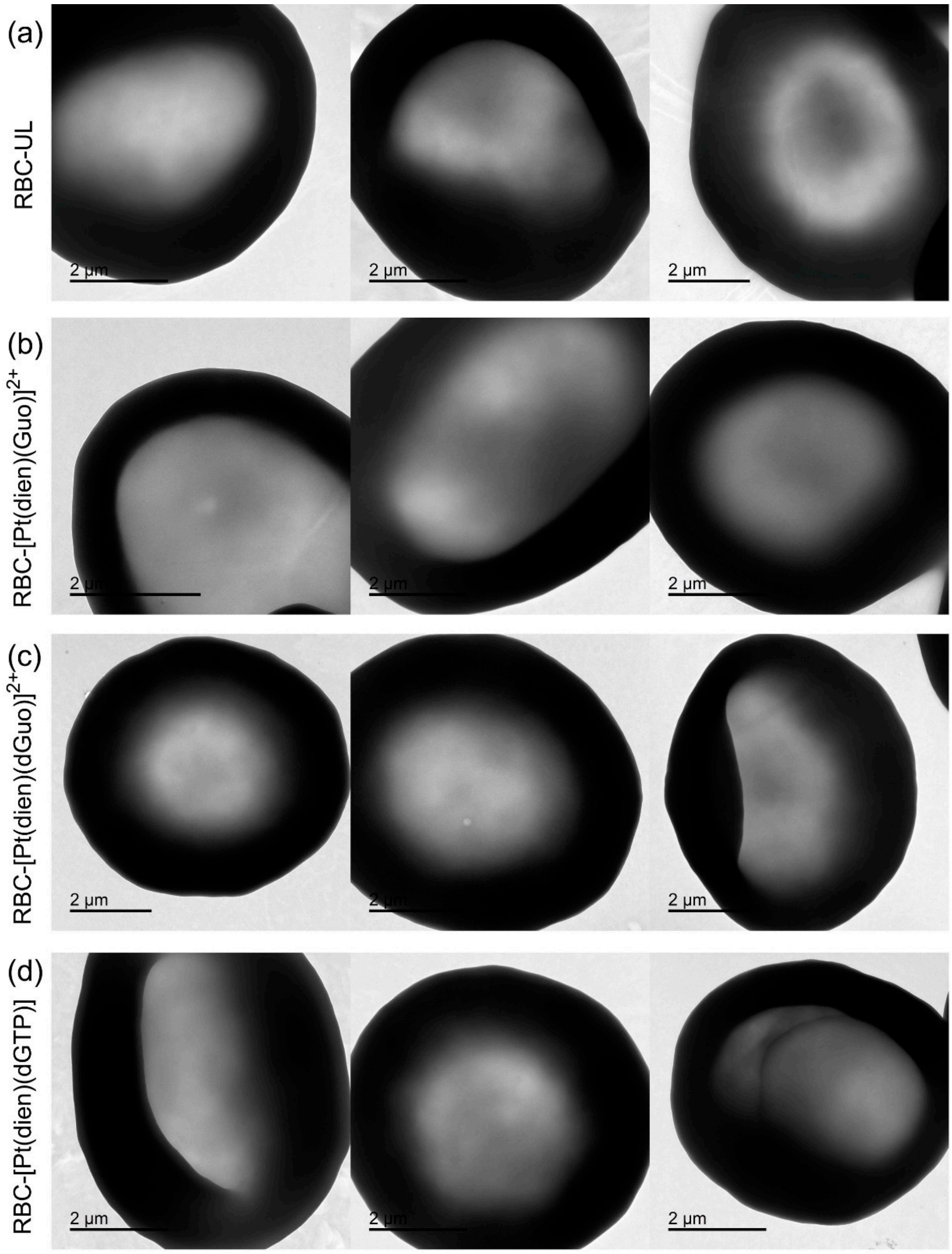

Figure S4. Transmission electron microscopy (TEM) analysis of: (a) Unloaded, (b) $[Pt^{II}(dien)(\textit{N7}\text{-}Guo)]^{2+}$- (c) $[Pt^{II}(dien)(\textit{N7}\text{-}dGuo)]^{2+}$-, and (d) $[Pt^{II}(dien)(\textit{N7}\text{-}dGTP)]$-loaded Red Blood Cells (dien = diethylenetriamine; Guo = guanosine; dGuo = 2'-deoxy-guanosine; dGTP = 5′-(2′-deoxy)-guanosine-triphosphate; in this figure for complexes **1-3** formulae the indication of platinum oxidation state and bonded *N7* have been omitted for simplicity).

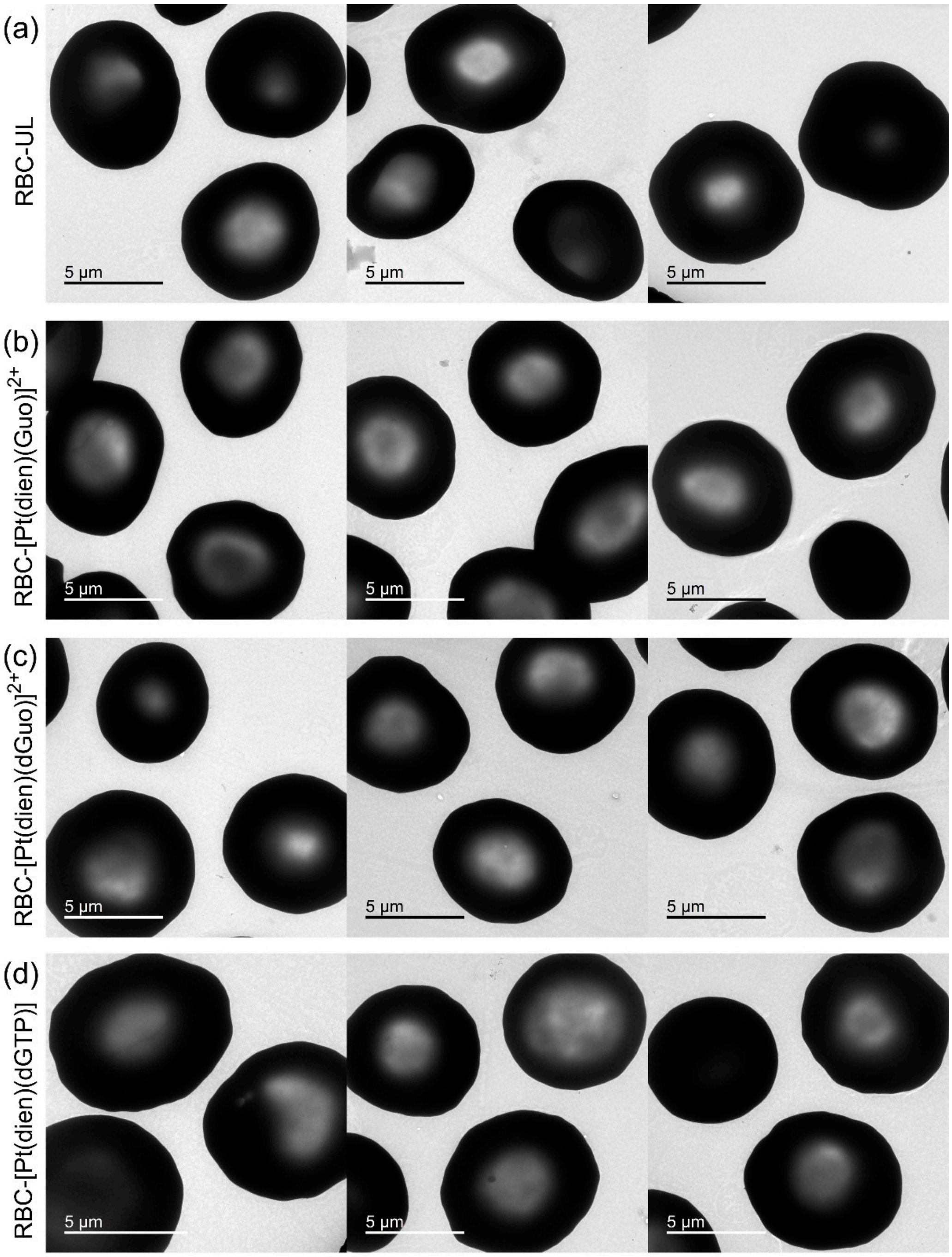

Figure S5. Transmission electron microscopy (TEM) analysis of: (a) Unloaded, (b) $[Pt^{II}(dien)(\mathit{N7}\text{-}Guo)]^{2+}$- (c) $[Pt^{II}(dien)(\mathit{N7}\text{-}dGuo)]^{2+}$-, and (d) $[Pt^{II}(dien)(\mathit{N7}\text{-}dGTP)]$-loaded Red Blood Cells (dien = diethylenetriamine; Guo = guanosine; dGuo = 2'-deoxy-guanosine; dGTP = 5′-(2′-deoxy)-guanosine-triphosphate; in this figure for complexes **1**-**3** formulae the indication of platinum oxidation state and bonded *N7* have been omitted for simplicity).
